# No efficacy of annual gynaecological screening in BRCA1/2 mutation carriers; an observational follow-up study

**DOI:** 10.1038/sj.bjc.6603725

**Published:** 2007-04-10

**Authors:** B B J Hermsen, R I Olivier, R H M Verheijen, M van Beurden, J A de Hullu, L F Massuger, C W Burger, C T Brekelmans, M J Mourits, G H de Bock, K N Gaarenstroom, H H van Boven, T M Mooij, M A Rookus

**Affiliations:** 1 Department of Obstetrics and Gynaecology, VU University Medical Centre, Amsterdam, The Netherlands; 2 Department of Gynaecology, The Netherlands Cancer Institute, Amsterdam, The Netherlands; 3 Department of Obstetrics and Gynaecology, Radboud University Nijmegen Medical Centre, Nijmegen, The Netherlands; 4 Departments of Obstetrics and Gynaecology, Erasmus University Medical Centre, Rotterdam, The Netherlands; 5 Department of Medical Oncology, Erasmus University Medical Centre, Rotterdam, The Netherlands; 6 Department of Obstetrics and Gynaecology, University Medical Centre Groningen, Groningen, The Netherlands; 7 Department of Epidemiology, University Medical Centre Groningen, Groningen, The Netherlands; 8 Department of Obstetrics and Gynaecology, Leiden University Medical Centre, Leiden, The Netherlands; 9 Department of Pathology, The Netherlands Cancer Institute, Amsterdam, The Netherlands; 10 Department of Epidemiology, The Netherlands Cancer Institute, Amsterdam, The Netherlands

**Keywords:** ovarian carcinoma, BRCA1, BRCA2, screening, TVU, CA125

## Abstract

BRCA1/2 mutation carriers are offered gynaecological screening with the intention to reduce mortality by detecting ovarian cancer at an early stage. We examined compliance and efficacy of gynaecological screening in BRCA1/2 mutation carriers. In this multicentre, observational, follow-up study we examined medical record data of a consecutive series of 888 BRCA1/2 mutation carriers who started annual screening with transvaginal ultrasonography and serum CA125 between 1993 and 2005. The women were annually screened for 75% of their total period of follow-up. Compliance decreased with longer follow-up. Five of the 10 incident cancers were interval tumours, diagnosed in women with a normal screening result within 3–10 months before diagnosis. No difference in stage distribution between incident screen-detected and interval tumours was found. Eight of the 10 incident cancers were stage III/IV (80%). Cancers diagnosed in unscreened family members had a similar stage distribution (77% in stage III/IV). The observed number of cases detected during screening was not significantly higher than expected (Standardized Incidence Ratio (SIR): 1.5, 95% confidence interval: 0.7–2.8). For the subgroup that was fully compliant to annual screening, a similar SIR was found (1.6, 95% confidence interval: 0.5–3.6). Despite annual gynaecological screening, a high proportion of ovarian cancers in BRCA1/2 carriers are interval cancers and the large majority of all cancers are diagnosed in advanced stages. Therefore, it is unlikely that annual screening will reduce mortality from ovarian cancer in BRCA1/2 mutation carriers.

BRCA1/2 mutation carriers have a high lifetime risk of developing ovarian cancer (39% for BRCA1, 11–22% for BRCA2 at age 70 years) (Antoniou *et al*, 2003; Chen *et al*, 2006). To reduce the mortality of ovarian cancer, BRCA1/2 mutation carriers are currently being counselled for gynaecological screening and prophylactic bilateral (salpingo-) oophorectomy (BP(S)O). Although efficacy of BPSO has been demonstrated (Rebbeck *et al*, 2005), the efficacy of gynaecological screening with (bi) annual transvaginal ultrasonography (TVU) and the serum tumour marker CA125 (Karlan *et al*, 1993; Burke *et al*, 1997) is still unclear. To summarize the literature on gynaecological screening of BRCA1/2 carriers, including overlap with the present study, eight interval cancers among 24 cancers were diagnosed among a total of 807 BRCA1/2 carriers (Table 1) (Laframboise *et al*, 2002; Liede *et al*, 2002; Scheuer *et al*, 2002; Fries *et al*, 2004; Kauff *et al*, 2005; Meeuwissen *et al*, 2005; Vasen *et al*, 2005; Gaarenstroom *et al*, 2006; Oei *et al*, 2006). Regretfully, most studies did not distinguish between prevalent and incident cases. Also, compliance to the intended screening protocol was generally unclear, whereas lack of compliance may interfere with the efficacy of screening.

Especially for BRCA1/2 carriers, more information on compliance and efficacy of gynaecological screening is urgently needed. Although compliance to the protocol may be higher for BRCA1/2 carriers than for other high-risk women, efficacy of screening may still be low. Ovarian tumours of carriers may have unfavourable clinical–pathological characteristics and a higher growth rate (Hogg and Friedlander, 2004). Moreover, carriers generally start screening well before menopause, when temporary abnormalities in TVU and CA125 for benign reasons are more common.

The purpose of this multicentre observational follow-up study was to investigate the efficacy of annual gynaecological screening taking compliance to the protocol into account. We compared numbers and stages at diagnoses of incident interval cancers with incident screen-detected cancers in a consecutive series of 888 BRCA1/2 carriers. In addition, we used two external comparisons: (1) ovarian cancers diagnosed in family members before gynaecological screening was available and, (2) BRCA1/2 reference curves for ovarian cancer.

## MATERIALS AND METHODS

### Study population

For this multicentre observational follow-up study, we identified a consecutive series of all BRCA1/2 mutation carriers who started gynaecological screening in six University Family Cancer Clinics in the Netherlands (VU University Medical Centre, The Netherlands Cancer Institute, Radboud University Nijmegen Medical Centre, Erasmus University Medical Centre Rotterdam, University Medical Centre Groningen, and Leiden University Medical Centre) in the period 1993–2005. In total, 1035 women with a deleterious BRCA1 or BRCA2 mutation visited the gynaecologists. The specific mutations were not known by the gynaecologists, but the prevalence of BRCA1/2 mutations in the Netherlands have been presented by Hout van der *et al* (2006). Women presenting with complaints at first visit and women who visited the gynaecologist only once were excluded. Among the remaining 888 women, five prevalent cancers were detected at the first screening visit, leaving 883 women (683 BRCA1 and 200 BRCA2) for the evaluation of screening during follow-up.

For analyses on compliance and sensitivity, we had to restrict the study population to women who visited one of the three centres (VU University Medical Centre, The Netherlands Cancer Institute and Radboud University Nijmegen Medical Centre) where information on each single screening visit (*N*=601 of the total group of 1035) was available, and to the 459 women who were ‘annually screened’, defined as having had at least one screening visit with both screening tools and another visit to create time of follow-up. Among the 459 women, two prevalent screen-detected cases were detected, leaving 457 women for evaluation of annual screening during follow-up.

### Gynaecological screening

Since 1993, the national screening guideline implied annual visits at the gynaecologist, including pelvic examination, TVU and serum CA125 analysis (www.nvog.nl). From the late nineties onward biannual visits have been introduced, though not systematically in all centres. For both BRCA1 and BRCA2 mutation carriers, the minimum age of entry into the surveillance programme was 35 years or 5 years earlier than the youngest age at diagnosis of ovarian cancer in the family.

TVU findings were classified as abnormal for ovaries or fallopian tubes, or normal including non-visualized ovaries. Serum CA125 levels above 35 kU l^−1^ were scored as abnormal if the clinical decision based on these findings was an extra-screening visit or a diagnostic operation (laparoscopy or laparotomy). Prophylactic operation that followed a visit within 3 months, while at this visit abnormalities were detected, was coded as diagnostic operation (*N*=8). Ovarian cancer cases were classified as prevalent screen-detected cases (diagnosed at the first screening visit), incident screen-detected cases (diagnosed at a regular screening visit) or as incident interval cases (diagnosed as a result of abdominal complaints, although no abnormalities were found at the previous screening visit). All tumours were reviewed by one pathologist (HvB).

### Data collection

The data for this study comprised BRCA1/2 mutation status, first and last gynaecological screening visits, prophylactic and diagnostic operation and pathology, as recorded in gynaecological medical records and pathological reports. In three centres, detailed data on each screening visit (date of visit, date of genetic testing, TVU, CA125, and reason to stop screening) were collected. Part of our pooled data was also used for earlier centre-specific publications (Table 1).

We used two external control groups. First, in two centres (Erasmus University Medical Centre Rotterdam and Netherlands Cancer Institute) pedigree information and the hospital-based cancer registry enabled us to trace anonymously tumours diagnosed in family members of the screened BRCA1/2 carriers. We evaluated those tumours diagnosed in the two centres before gynaecological screening started (before 1990). Second, we used BRCA1/2 reference curves of ovarian cancer derived as part of refitting the BOADICEA model of genetic susceptibility to breast cancer (Antoniou *et al*, 2002; Antoniou *et al*, 2004). Combined data were used from three UK population-based studies of breast cancer families (Peto *et al*, 1999; Angelian Breast Cancer Study Group, 2000; Lalloo *et al*, 2003), with multiple cases of breast cancer and family data from BRCA1/2 carriers identified in 22 population-based studies of breast and ovarian cancer (Antoniou *et al*, 2003).

### Statistical analysis

To calculate the Standardized Incidence Ratio (SIR), observed numbers of ovarian cancer were compared with expected numbers, based on the reference curves. Women were eligible for person-years analysis if no ovarian cancer was detected at the first screening visit. Starting date was defined as date of first visit, stopping date as date of diagnosis of ovarian cancer (end point), date of BP(S)O, or date of last screening visit, whichever was first. In the three centres with information on each single screening visit, the period of optimal annual screening was defined as the period during which the national guideline (‘complete screening visit’, i.e. visit with both TVU and CA125, every 13 months) was met. Here, stopping date was defined as date of diagnosis of ovarian cancer, date of BP(S)O, date of last complete screening visit plus 13 months, or date of last visit, whichever was first.

Women who decide to be tested may be more likely to have ovarian cancer than those who are not yet aware of their carrier status and, thus, are not eligible for the study (Klaren *et al*, 2003). If cancer events preceding the DNA test are included in the analysis, overestimation of the incidence rate may occur. We explored this potential testing bias by starting follow-up at date of first complete screening visit or date of BRCA1/2 mutation testing, whichever was later.

## RESULTS

**Figure 1:**
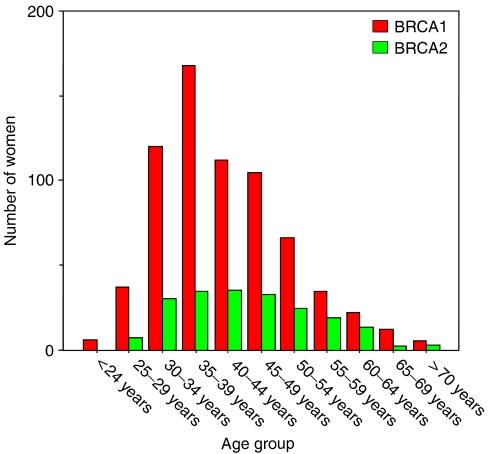
Age distribution at first visit of 683 BRCA1 mutation carriers compared with 200 BRCA2 mutation carriers.

In total, 1035 BRCA1/2 carriers ever visited a gynaecologist for screening advice and 883 BRCA1/2 carriers were actually screened for 1473 women-years of follow-up. At first visit, the 683 BRCA1 carriers (77%, median age 40 years, range 21–76 years) were on average 3 years younger than the 200 BRCA2 carriers (23%, median age 44 years, range 25–77 years, Figure 1).

### Compliance and quality of screening tools

Among the 601 women with full data on each single visit, 118 women (19.6%) visited the Family Cancer Clinic only once (Table 2), whereas 24 women with more visits were never screened with both TVU and CA125. Most women opted out of screening for valid reasons, like being too young (median age 28 years, range 20–34 years) or undergoing prophylactic operation (median age 44 years, range 26–75 years). Thus, the default rate was only 31/601=5% in the total group, and 6% including the women with missing data. Still, however, for 25% of the screening time (interval between starting and stopping dates) the interval between two complete screening visits was more than 13 months. This proportion of non-compliance increased with longer follow-up.

The sensitivity of TVU and CA125 assessed simultaneously was 71%, and the positive predictive value 23% (Table 3). As shown in Table 2, 311 of the 459 women (68%) opted for a BP(S)O and five occult tumours were diagnosed. Including these tumours, the sensitivity decreased to 42%.

### Cases of ovarian cancer

**Figure 2:**
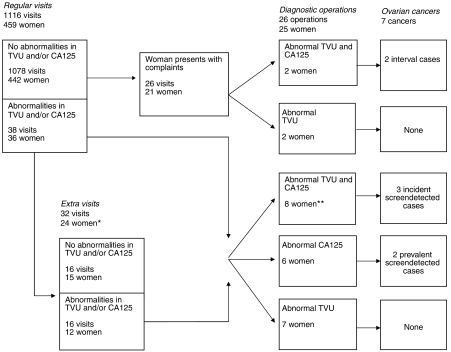
Flow diagram of the screening process. ^*^ Eight women had extra visits during follow-up. ^**^One woman underwent diagnostic operation twice.

At the first screening visit, five women (four BRCA1 and one BRCA2) were diagnosed with a prevalent ovarian cancer (Table 4). Among the remaining 883 BRCA1/2 carriers 10 women (all BRCA1) were diagnosed with ovarian cancer during follow-up (incident cases). Nine of the 10 incident cases had been compliant to annual screening before diagnosis. In an efficiently screened population, the majority of cancers would be detected by screening instead of diagnosed following complaints. However, in our series five of 10 incident cancers were unexpectedly diagnosed as a result of abdominal complaints, although at the preceding visit no abnormalities had been found. The five incident screen-detected cases presented with both an abnormal TVU and CA125, although no abnormalities were detected at the preceding visit (Table 4, Figure 2). The family history of the 10 incident cases varied, three cases had a breast-only family, one case an ovarian-only family and six cases had both familial breast and familial ovarian cancer.

**Figure 3:**
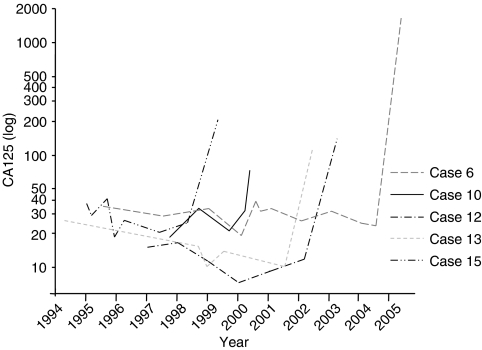
CA125 levels of women with ovarian cancer detected at regular screening visits (nos. 12, 13 and 15) or detected in between two visits (i.e. interval ovarian cancer cases; 6 and 10); numbers of cases consistent with Table 4.

Seven cases (indicated in bold in Table 4) were diagnosed in the group of 459 women that underwent annual screening in one of the three centres with information on each screening visit. These 459 women came for 1116 screening visits during 690 annually screened women-years (2.4 visits/woman and 1.6 visits/year) (Figure 2). Abnormalities were detected in one or both of the screening tools, at 38 out of 1116 regular screening visits (3%). For 24 women (5%), the abnormal findings were followed by 32 extra visits at which 16 abnormalities persisted (50%). After normal regular screening visits, complaints resulted in 26 visits for 21 women (5%) at which four abnormalities were found (15%). In total, abnormalities were found in 40 women (9%) resulting in 26 diagnostic operations. No cancer was detected at nine operations that followed abnormal TVU only, two cancers were detected at six operations that followed abnormal CA125 only (33%), and five cancers were detected at 11 operations (45%) that followed abnormal findings in both screening tools. Compared to the next to last visit, an exponential rather than progressive rise in serum CA125 levels from the individual baseline level occurred at the last visit for all incident screen-detected and interval cases (Figure 3).

### Stages at diagnoses: interval *vs* screen-detected cancers

Advanced stages (III/IV) were diagnosed in all prevalent screen-detected cases. For the incident screen-detected cases, the stages at diagnosis were stage II (*N*=1), stage III (*N*=3) and stage IV (*N*=1), whereas for the incident interval cases, tumours were diagnosed at stage II (*N*=1), stage III (*N*=2) and stage IV (*N*=2). At a mean follow-up since diagnosis of 28 months, three of the 15 cases listed in Table 4 died of ovarian cancer. The five occult tumours detected at BP(S)O (Table 2) were diagnosed at stage I (*N*=4) and stage II (*N*=1). Premalignant lesions in prophylactically removed tissue were not the scope of this study, but were subject of separate hospital-based studies (e.g. Hermsen *et al*, 2006). In none of the women who were considered normal at surgery, evidence of tumour growth occurred during follow-up, so far.

### Stages at diagnoses: comparison with cancers in unscreened family members

If screening were effective, ovarian cancer would be diagnosed at an earlier stage with better prognosis than would have been the case without screening. We evaluated this potential shift in stage by comparing the stages at diagnosis of the incident screen-detected tumours with tumours diagnosed in family members before gynaecological screening was available. In two centres, we could trace 26 family members with ovarian cancer diagnosed before 1990. Stages at diagnosis were stage I (*N*=3), stage II (*N*=3), stage III (*N*=19) and stage IV (*N*=1). For the subgroup of typed or obligate carriers (*N*=16), the numbers were: stage I (*N*=2), stage II (*N*=2), stage III (*N*=12) and stage IV (*N*=0). Thus, the stage distributions of the screened and unscreened groups were similar (stage III/IV: eight in 10 (80%) in the screened group *vs* 20 in 26 (77%) in the unscreened group).

### Comparison with external reference curves

If screening were effective, the diagnosis would shift to an earlier age. We evaluated the lead-time by comparing the observed number of cases with the number expected from age- and mutation-specific external incidence curves, as estimated in an international data set, comprising women that most likely were less intensely screened (Antoniou *et al*, 2003). Given the rising incidence curve at the young ages of the screened group, one would expect the lead time to result in an increased SIR. However, based on 10 incident cases observed and 6.5 cases expected, the SIR was only 1.5 (95% confidence Interval (CI) 0.7–2.8) (Table 5). Among BRCA1 carriers, the SIR was 1.7 (95% CI: 0.8–3.1) and among BRCA2 carriers the SIR could not be estimated with no event observed and 0.5 expected. The SIR was 1.6 (95% CI 0.5–3.6) if the analysis was restricted to the optimally screened women-years. As only one case was tested for BRCA1/2 mutation after the diagnosis of ovarian cancer, testing bias did not markedly confound the results (4% change of the SIR, data not shown).

## DISCUSSION

We evaluated the actual gynaecological screening in a consecutive series of 888 BRCA1/2 mutation carriers. Although compliance was reasonably high (compliant for 75% of the follow-up), gynaecological screening did not seem to be effective, because (1) interval tumours comprised five out of 10 incident cancers, (2) all women diagnosed with an interval tumour had been compliant to annual screening, (3) eight of the 10 cancers were diagnosed at stage III/IV, (4) no difference in stage distribution between incident screen-detected and interval tumours was found, (5) no difference in stage distribution between incident screen-detected tumours and tumours diagnosed in family members before 1990 was found, and (6) in the total group as well as in the compliant group the observed number of ovarian cancers was not markedly higher than expected from reference curves, based on carriers of which the majority was most likely not screened. Thus, although we could not formally test efficacy with mortality as outcome, it is unlikely that annual gynaecological screening will reduce mortality of ovarian cancer in BRCA1/2 carriers.

Reported compliance of high-risk women to gynaecological screening proved to vary extremely among studies (Lerman *et al*, 2000; Scheuer *et al*, 2002; Botkin *et al*, 2003; Vasen *et al*, 2005). This may result from differences in definition of compliance, differences in the requested frequency of screening visits and differences in risk perception of the women and their physicians (Tinley *et al*, 2004). For instance, in a series of 112 high-risk women (29% BRCA1/2 carriers) adherence to annual TVUs was only 19%, whereas in a group of 62 BRCA1/2 carriers adherence was 68% to biannual screening with CA125 (Scheuer *et al*, 2002). We found that women were compliant for 75% of their follow-up, but valid reasons like pregnancies or breast cancer treatments may partly explain why these BRCA1/2 carriers missed or delayed their annual screening visits.

TVU and serum CA125 lack adequate sensitivity apart or together (15–71% for TVU/CA125) (Laframboise *et al*, 2002; Scheuer *et al*, 2002; Tailor *et al*, 2003; Meeuwissen *et al*, 2005; Stirling *et al*, 2005; Olivier *et al*, 2006) and our finding of 42% for TVU/CA125 is in accordance with these findings.

The external reference population that we used to calculate the expected number of ovarian cancer, consisted of family members of a largely population-based index case series, who were tested positive in a research setting. Most of these women may not have been aware of their carrier status and thus, might be less intensively screened than the BRCA1/2 carriers in our study. However, this assumption is not the only reason why the estimated SIR should be interpreted with caution. Oral contraceptives strongly protect against ovarian cancer (Antoniou *et al*, 2003; Whittemore *et al*, 2004), and use of oral contraceptives might differ between our study sample and the reference population. With these limitations in mind, the SIRs suggest that the lead-time is very limited in the total and compliant group.

In evaluating efficacy of screening, several factors hamper the comparison among various studies, (1) compliance to the intended screening procedure has not been examined in combination with efficacy, (2) generally, no distinction is made between prevalent and incident screen-detected cases, (3) the screening protocol may differ, such as the frequency of screening, the cutoff level of CA125 (15–35 U ml^−1^) (Bourne *et al*, 1994; Jacobs *et al*, 1999; Kauff *et al*, 2005; Meeuwissen *et al*, 2005; Olivier *et al*, 2006), and the combined or sequential order of applying the screening tools (Jacobs *et al*, 1999; Scheuer *et al*, 2002; Meeuwissen *et al*, 2005; Stirling *et al*, 2005; Olivier *et al*, 2006), and (4) quality measures of screening tools, like sensitivity, are typically reported including occult tumours, while the proportion of women opting for a BP(S)O differs strongly across various countries (Wainburg and Husted, 2004). Consequently, the proportion of interval cancers detected during screening varies among studies from 5/7=71% in the study by Liede *et al* (2002), 1/3=33% in the study by Scheuer *et al* (2002) and 1/6=17% in the study by Vasen *et al* (2005) (Table 1). In our study, the proportion of interval cancers was 5/15=33% including the prevalent screen-detected cases and 5/10=50% excluding the prevalent cases. Apart from this high proportion of interval cancers, the unfavourable stages at diagnoses for incident screen-detected cancers as well as for interval cancers (stage III/IV: eight out of 10 cases in our study, even excluding prevalent screen-detected cases) were quite disappointing given the high compliance of cases. Moreover, the stage distribution in our historical cases supported the lack of a shift to lower stages by the introduction of screening. In other studies, the stage distribution may be more (Scheuer *et al*, 2002) or less (Liede *et al*, 2002) favourable, but power of most studies was very low (Table 1).

In conclusion, annual gynaecological screening with TVU and CA125 does not seem to be effective for BRCA1/2 carriers. In our series, all but one case were diagnosed as a result of an abnormal CA125 with or without an abnormal TVU. Thus, the question remains, whether more frequent CA125 measurements would have given better results. Seven of the 10 incident cases were diagnosed 9–14 months after the last visit with normal findings, suggesting room for improvement. However, the three incident cases that were diagnosed within 6 months of the last visit with normal findings were all diagnosed at stage III/IV. Therefore, our limited data on this issue do not suggest that the efficacy of gynaecological screening will be significantly improved by more frequent screening with CA125, although evidence should come from trials. Hopefully, the search for other tumour markers, for instance by proteomics, will generate more promising alternatives. In a case–control study, early stage ovarian cancer could be detected by taking three new biomarkers into account. The sensitivity of the combination of apolipoprotein A1, transthyretin, H4 and CA125 was higher than CA125 alone (Zhang *et al*, 2004). A combination of four other proteins was identified with microarray analysis (Mor *et al*, 2005). However, clinical implementation of such early findings will need confirmatory and prospective studies in larger groups. In the general population, there are two major randomised controlled trials being undertaken to assess the impact of screening on ovarian cancer mortality; the UK Collaborative Trial of Ovarian Cancer Screening (UKCTOCS) and the Prostate, Lung, Colorectal and Ovarian (PLCO) Cancer Screening Trial in the United States (Andriole *et al*, 2004; Menon, 2004, respectively).

For now, it is unlikely that annual gynaecological screening with TVU and CA125 will reduce mortality from ovarian cancer in BRCA1/2 mutation carriers. Prophylactic removal of the ovaries and fallopian tubes has proven its value as a risk-reducing strategy for ovarian cancer as well as breast cancer (Kauff *et al*, 2002; Rebbeck *et al*, 2002; Finch *et al*, 2006) and should therefore be the cornerstone in the management of BRCA1/2 mutation carriers, as long as no other effective screening tool is available.

## Figures and Tables

**Table 1:** Literature overview of studies concerning ovarian cancer detection in women at hereditary high risk and carriers of the BRCA1/2 mutation

				**Intended screening**			**Number of screen-detected**		**Number of interval cases**			**Number of screen-detected**		**Number of interval cases**	
**Author**	**Number of women^a^**	**Mean age (years)**	**Women years**	**Frequency**	**Combined/sequential TVU+CA-125 (cutoff)**	**Mean follow-up (months)**	**Stage I/II**	**Stage III/IV**	**Stage I/II**	**Stage III/IV**	**Number of carriers**	**Stage I/II**	**Stage III/IV**	**Stage I/II**	**Stage III/IV**
Laframboise *et al*, 2002	311	47	NR	Biannual	Combined (<35 kU ml^−1^)	NR	1/0	0/0	0/0	0/0	31	1/?0	0/0	0/0	0/0
Liede *et al*, 2002	290	42	1439	Biannual until 1995	Combined (<35 kU ml^−1^)	60	1/0	2/0	0/1	4/0	33	0/0	2/0	0/1	4/0
Scheuer *et al*, 2002	62	48	129	Biannual	Combined (cutoff NR)	25	2/1^a^	0/0	1/0	0/0	62	2/1^a^ 1	0/0	1/0	0/0
							1 not staged					not staged			
Fries *et al*, 2004	46	53	165	Twice annual	Combined (cutoff NA)	43	0/0	0/0	0/0	0/0	7	0/0	0/0	0/0	0/0
Vasen *et al*, 2005^c^	138	NR	426	Annual	Combined (cutoff NR)	37	0/0	0/5^b^	0/0	1/0	95	0/0	0/5^b^	0/0	1/0
Meeuwissen *et al*, 2005^c^	383	42	989	Biannual after 1998	Combined (<35 kU ml^−1^)	31	0/0	0/0	0/0	0/0	152	0/0	0/0	0/0	0/0
Kauff *et al*, 2005	135	49	225	Biannual	Combined (<35 kU ml^−1^)	20	0/0	0/0	0/0	0/0	49	0/0	0/0	0/0	0/0
Oei *et al*, 2006^c^	512	42	1029	Annual	Combined (<35 kU ml^−1^)	25	0/0	1/0	0/0	0/0	265	0/0	1/0	0/0	0/0
Gaarenstroom *et al*, 2006^c^^,^^d^	269	45	583	Annual	Combined (<35 kU ml^−1^)	26	1/0	4/0	0/0	1/1	113	0/0	3/0	0/0	0/1
															
Total	2146	44	4985			28	5/1	7/5	1/1	6/1	807	2 or 3/1	6/5	1/1	5/1
							1 not staged								

NR=not reported.

a Two of these cases had abnormal TVU at first screening and could be defined as prevalent screen-detected cases.

b Two of these cases were prevalent screen-detected cases, for other studies, numbers of prevalent screen-detected cases were not indicated.

c Study that (partly) overlaps with present pooled study.

d Three of these cases were prevalent screen-detected cases.

**Table 2:** Reasons not to start or to stop screening (at least annual screening with TVU and CA-125) for all 601 BRCA1/2 mutation carriers who visited the gynaecologist at the Family Cancer Clinic in three centres

	**With follow-up**				**Without follow-up**		**Total number**
	**Screened with both TVU and CA-125**		**Screened but never with both TVU and CA-125**				
**Status at end of follow-up**	***N***	**%**	***N***	**%**	***N***	**%**	***N***
In screening	97	21.1	4	16.6	9	7.6	110
Did not show up at appointment	14	3.1	4	16.6	13	11.0	31
Moved to other place/other gynaecologist	7	1.5	2	8.3	21	17.8	30
Too young	3	0.7	4	16.6	42	35.6	49
Too old	1	0.2	0	0	2	1.7	3
Prophylactic operation^a^	311	67.8	10	41.7	28	23.7	349
Missing	1	0.2	0	0	3	2.5	4
Diagnostic operation	25^b^	5.4	0	0	0	0	25
							
Total	459	100	24	100	118	100	601

a Five occult tumours were detected (4 stage I, 1 stage II).

b One woman had two diagnostic operations.

**Table 3:** Quality of screening tools used during combined multimodal gynaecological screening of 459 BRCA1/2 carriers during 1116 regular screening visits

**Test**	**Number of cases^a^**	**Number of unaffected women**	**Sensitivity (95% CI)**	**Specificity (95% CI)**	**Positive predictive value (95% CI)**	**Negative predictive value (95% CI)**
*CA-125, irrespective of TVU*						
+	5 (*5)*	10 (*10)*	42% (14–70)	99% (99–100)	33% (9–57)	99% (99–100)
−	7 (*2)*	1094 (*1099)*	*71*% (*38–100)*	*99*% (*99–100)*	*33*% (*9–57)*	*100*% (*100–100)*
						
*TVU, irrespective of CA-125*						
+	3 (*3)*	12 (*12)*	25% (1–50)	99% (98*–*100)	20% (0–40)	99% (99–100)
−	9 (*4)*	1092 (*1097)*	*43*% (*6–80)*	*99% (98–100)*	*20*% (*0–40)*	*100*% (*99–100)*
						
*CA-125 and/or TVU*						
+	5 (*5)*	17 (*17)*	42% (14–70)	99% (98*–*99)	23% (5*–*40)	99% (99–100)
−	7 (*2)*	1087 (*1092*)	*71*% (*38–100*)	*99*% (*98–99)*	*23*% (*5–40*)	*100*% (*100–100)*

CI, confidence interval.

a In italic: numbers and percentages without the five occult ovarian cancer cases, diagnosed during prophylactic operation, were included in the case group.

**Table 4:** Cases diagnosed with ovarian cancers among 888 BRCA1/2 carriers during gynaecological screening

		**Second last screening visit**			**Last screening visit^a^**							
	**DNA**	**TVU**	**CA-125 (kU ml^−1^)**	**Interval between visits (months)**	**TVU**	**CA-125 (kU ml^−1^)**	**Detection**	**Age at diagnosis**	**Histological classification (grade)**	**FIGOStage**	**Status**	**Follow-up after diagnosis (months)**
1	BRCA2	NA	NA	NA	Normal	331	Prevalent screen-detected	77	Endometrioid (GIII)	IIIB	NED	31
**2**	**BRCA1**	**NA**	**NA**	**NA**	**Normal**	**113**	**Prevalent screen-detected**	**52**	**Serous (GIII)**	**IIIC**	**NED**	**5**
3	BRCA1	NA	NA	NA	Abnormal	188	Prevalent screen-detected	56	Serous (GIII)	IIIC	SD	16
4	BRCA1	NA	NA	NA	Normal	211	Prevalent screen-detected	47	Serous (GII)	IIIC	NED	5
**5**	**BRCA1**	**NA**	**NA**	**NA**	**Normal**	**138**	**Prevalent screen-detected**	**45**	**Serous (GIII)**	**IV**	**RE**	**11**
												
**6**	**BRCA1**	**Normal**	**22**	**10**	**Abnormal**	**1693**	**Incident interval**	**59**	**Endometrioid (GIII)**	**IIC**	**NED**	**3**
7	BRCA1	Normal	15	4	Abnormal	15	Incident interval	73	Serous (GIII)	IIIC	NED	43
8	BRCA1	Normal	5	10	Abnormal	1450	Incident interval	38	Mucinous (GIII)	IIIC	SD	48
9	BRCA1	Normal	15	9	Abnormal	202	Incident interval	39	Serous (only cytology)	IV	SD	19
**10**	**BRCA1**	**Normal**	**33**	**3**	**Abnormal**	**82**	**Incident interval**	**33**	**Mucinous (GI)**	**IV**	**DOD**	**19**
												
11	BRCA1	Normal	11	14	Abnormal	1815	Incident screen-detected	43	Endometrioid (GII)	IIC	NED	34
**12**	**BRCA1**	**Normal**	**12**	**13**	**Abnormal**	**159**	**Incident screen-detected**	**55**	**Serous (GIII)**	**IIIB**	**NED**	**28**
**13**	**BRCA1**	**Normal**	**10**	**11**	**Abnormal**	**105**	**Incident screen-detected**	**50**	**Endometrioid (GIII)**	**IIIB**	**DOD**	**39**
14	BRCA1	Normal	11	6	Abnormal	48	Incident screen-detected	73	Serous (GIII)	IIIB	DOD	41
**15**	**BRCA1**	**Normal**	**24**	**12**	**Normal**	**217**	**Incident screen-detected**	**52**	**Serous (GIII)**	**IV**	**NED**	**75**

DOD=death of disease; NA=not applicable; NED=no evidence of disease; RE=recurrence of disease; SD=stable disease.

In bold: women diagnosed in one of the three centres with information on each screening visit.

a Last visit is last screenings visit for the interval and incident screen-detected cases.

**Table 5:** SIR calculated for the total group of 883 BRCA1/2 mutation carriers and for the subgroup of 457 BRCA1/2 mutation carriers, known to be annually screened^a^

**Women-years**	**Number of women**	**Number of women-years**	**Observed**	**Expected^b^**	**SIR (95% Confidence Interval)**
All women-years, six centres	883	1473	10	6.5	1.5 (95% CI: 0.7–2.8)
All women-years, three centres	457	921	5	3.9	1.3 (95% CI: 0.4–3.0)
Exclusively annually screened women-years, three centres	457	690	5	3.2	1.6 (95% CI: 0.5–3.6)

CI, confidence interval; SIR=Standardized Incidence Rates.

a In three of the six centres, data were available for each screening visit, which enabled the selection of women who were at least some time annually screened, that is, a visit with both TVU and CA125 and some time of follow-up.

b Reference data: age- and mutation-specific risk of ovarian cancer among BRCA1/2 mutation carriers, as modelled by Antoniou *et al* (2002, 2004).

## References

[bib1] Andriole GL, Reding D, Hayes RB, Prorok PC, Gohagan JK (2004) The prostate, lung, colon, and ovarian (PLCO) cancer screening trial: status and promise. Urol Oncol 22(4): 358–361 15283897 10.1016/j.urolonc.2004.04.013

[bib2] Anglian Breast Cancer Study Group (2000) Prevalence and penetrance of BRCA1 and BRCA2 mutations in a population-based series of breast cancer cases. Br J Cancer 83(10): 1301–1308 11044354 10.1054/bjoc.2000.1407 PMC2408797

[bib3] Antoniou A, Pharoah PD, Narod S, Risch HA, Eyfjord JE, Hopper JL, Loman N, Olsson H, Johannsson O, Borg A, Pasini B, Radice P, Manoukian S, Eccles DM, Tang N, Olah E, Anton-Culver H, Warner E, Lubinski J, Gronwald J, Gorski B, Tulinius H, Thorlacius S, Eerola H, Nevanlinna H, Syrjakoski K, Kallioniemi OP, Thompson D, Evans C, Peto J, Lalloo F, Evans DG, Easton DF (2003) Average risks of breast and ovarian cancer associated with BRCA1 or BRCA2 mutations detected in case series unselected for family history: a combined analysis of 22 studies. Am J Hum Genet 72(5): 1117–1130 12677558 10.1086/375033 PMC1180265

[bib4] Antoniou AC, Pharoah PD, McMullan G, Day NE, Stratton MR, Peto J, Ponder BJ, Easton DF (2002) A comprehensive model for familial breast cancer incorporating BRCA1, BRCA2 and other genes. Br J Cancer 86(1): 76–83 11857015 10.1038/sj.bjc.6600008 PMC2746531

[bib5] Antoniou AC, Pharoah PDP, Easton DF, on behalf of the Boadicea Collaborators (2004) The BOADICEA model of genetic susceptibility to breast and ovarian cancer: updating and validation. Annual Meeting of the American Society of Human Genetics poster abstract 555

[bib6] Botkin JR, Smith KR, Croyle RT, Baty BJ, Wylie JE, Dutson D, Chan A, Hamann HA, Lerman C, McDonald J, Venne V, Ward JH, Lyon E (2003) Genetic testing for a BRCA1 mutation: prophylactic surgery and screening behavior in women 2 years post testing. Am J Med Genet 118(3): 201–209 10.1002/ajmg.a.10102 12673648

[bib7] Bourne TH, Campbell S, Reynolds K, Hampson J, Bhatt L, Crayford TJ, Whitehead MI, Collins WP (1994) The potential role of serum CA 125 in an ultrasound-based screening program for familial ovarian cancer. Gynecol Oncol 52(3): 379–385 8157195 10.1006/gyno.1994.1065

[bib8] Burke W, Daly M, Garber J, Botkin J, Kahn MJ, Lynch P, McTiernan A, Offit K, Perlman J, Petersen G, Thomson E, Varricchio C (1997) Recommendations for follow-up care of individuals with an inherited predisposition to cancer. II. BRCA1 and BRCA2. Cancer genetics studies consortium. JAMA 277(12): 997–1003 9091675

[bib9] Chen S, Iversen ES, Friebel T, Finkelstein D, Weber BL, Eisen A, Peterson LE, Schildkraut JM, Isaacs C, Peshkin BN, Corio C, Leondaridis L, Tomlinson G, Dutson D, Kerber R, Amos CI, Strong LC, Berry DA, Euhus DM, Parmigiani G (2006) Characterization of BRCA1 and BRCA2 mutations in a large United States sample. J Clin Oncol 24(6): 863–871 16484695 10.1200/JCO.2005.03.6772 PMC2323978

[bib10] Finch A, Beiner M, Lubinski J, Lynch HT, Moller P, Rosen B, Murphy J, Ghadirian P, Friedman E, Foulkes WD, Kim-Sing C, Wagner T, Tung N, Couch F, Stoppa-Lyonnet D, Ainsworth P, Daly M, Pasini B, Gershoni-Baruch R, Eng C, Olopade OI, McLennan J, Karlan B, Weitzel J, Sun P, Narod SA (2006) Salpingo-oophorectomy and the risk of ovarian, fallopian tube, and peritoneal cancers in women with a BRCA1 or BRCA2 Mutation. JAMA 296(2): 185–192 16835424 10.1001/jama.296.2.185

[bib11] Fries MH, Hailey BJ, Flanagan J, Licklider D (2004) Outcome of five years of accelerated surveillance in patients at high risk for inherited breast/ovarian cancer: report of a phase II trial. Mil Med 169(6): 411–416 15281667 10.7205/milmed.169.6.411

[bib12] Gaarenstroom KN, Van der Hiel B, Tollenaar RAEM, Vink GR, Jansen FW, Van Asperen CJ, Kenter GG (2006) Efficacy of screening women at high risk of hereditary ovarian cancer: results of an 11-year cohort study. Int J Gynecol Cancer 16(Suppl 1): 54–59 16515568 10.1111/j.1525-1438.2006.00480.x

[bib13] Hermsen BBJ, Diest van PJ, Berkhof J, Menko FH, Gille JJP, Piek JMJ, Meijer S, Winters HAH, Kenemans P, Mensdorff-Pouilly von S, Verheijen RHM (2006) Low prevalence of (pre) malignant lesions in the breast and high prevalence in the ovary and Fallopian tube in women at hereditary high risk of breast and ovarian cancer. Int J Cancer 119(6): 1412–1418 16615107 10.1002/ijc.21988

[bib14] Hogg R, Friedlander M (2004) Biology of epithelial ovarian cancer: implications for screening women at high genetic risk. J Clin Oncol 22(7): 1315–1327 15051780 10.1200/JCO.2004.07.179

[bib15] Hout van der AH, Ouweland van den AM, Luijt van der RB, Gille HJ, Bodmer D, Bruggenwirth H, Mulder IM, Vlies van der P, Elfferich P, Huisman MT, Berge ten AM, Kromosoeto J, Jansen RP, Zon van PH, Vriesman T, Arts N, Boutmy-de Lange M, Oosterwijk JC, Meijers-Heijboer H, Ausems MG, Hoogerbrugge N, Verhoef S, Halley DJ, Vos YJ, Hogervorst F, Ligtenberg M, Hofstra RM (2006) A DGGE system for comprehensive mutation screening of BRCA1 and BRCA2: application in a Dutch cancer clinic setting. Hum Mutat 27(7): 654–666 16683254 10.1002/humu.20340

[bib16] Jacobs IJ, Skates SJ, MacDonald N, Menon U, Rosenthal AN, Davies AP, Woolas R, Jeyarajah AR, Sibley K, Lowe DG, Oram DH (1999) Screening for ovarian cancer: a pilot randomised controlled trial. Lancet 353(9160): 1207–1210 10217079 10.1016/S0140-6736(98)10261-1

[bib17] Karlan BY, Raffel LJ, Crvenkovic G, Smrt C, Chen MD, Lopez E, Walla CA, Garber C, Cane P, Sarti DA, Rotter JI, Platt LD (1993) A multidisciplinary approach to the early detection of ovarian carcinoma: rationale, protocol design, and early results. Am J Obstet Gynecol 169(3): 494–501 8372851 10.1016/0002-9378(93)90607-k

[bib18] Kauff ND, Hurley KE, Hensley ML, Robson ME, Lev G, Goldfrank D, Castiel M, Brown CL, Ostroff JS, Hann LE, Offit K, Barakat RR (2005) Ovarian carcinoma screening in women at intermediate risk: impact on quality of life and need for invasive follow-up. Cancer 104(2): 314–320 15948173 10.1002/cncr.21148

[bib19] Kauff ND, Satagopan JM, Robson ME, Scheuer L, Hensley M, Hudis CA, Ellis NA, Boyd J, Borgen PI, Barakat RR, Norton L, Offit K (2002) Risk-reducing salpingo-oophorectomy in women with a BRCA1 or BRCA2 mutation. N Engl J Med 346(21): 1609–1615 12023992 10.1056/NEJMoa020119

[bib20] Klaren HM, van’t Veer LJ, van Leeuwen FE, Rookus MA (2003) Potential for bias in studies on efficacy of prophylactic surgery for BRCA1 and BRCA2 mutation. J Natl Cancer Inst 95(13): 941–947 12837830 10.1093/jnci/95.13.941

[bib21] Laframboise S, Nedelcu R, Murphy J, Cole DE, Rosen B (2002) Use of CA-125 and ultrasound in high-risk women. Int J Gynecol Cancer 12(1): 86–91 11860541 10.1046/j.1525-1438.2002.01055.x

[bib22] Lalloo F, Varley J, Ellis D, Moran A, O’Dair L, Pharoah P, Evans DG (2003) Prediction of pathogenic mutations in patients with early-onset breast cancer by family history. Lancet 361(9363): 1101–1102 12672316 10.1016/S0140-6736(03)12856-5

[bib23] Lerman C, Hughes C, Croyle RT, Main D, Durham C, Snyder C, Bonne A, Lynch JF, Narod SA, Lynch HT (2000) Prophylactic surgery decisions and surveillance practices one year following BRCA1/2 testing. Prev Med 31(1): 75–80 10896846 10.1006/pmed.2000.0684

[bib24] Liede A, Karlan BY, Baldwin RL, Platt LD, Kuperstein G, Narod SA (2002) Cancer incidence in a population of Jewish women at risk of ovarian cancer. J Clin Oncol 20(6): 1570–1577 11896106 10.1200/JCO.2002.20.6.1570

[bib25] Meeuwissen PA, Seynaeve C, Brekelmans CT, Meijers-Heijboer HJ, Klijn JG, Burger CW (2005) Outcome of surveillance and prophylactic salpingo-oophorectomy in asymptomatic women at high risk for ovarian cancer. Gynecol Oncol 97(2): 476–482 15863147 10.1016/j.ygyno.2005.01.024

[bib26] Menon U (2004) Ovarian cancer screening. CMAJ 171(4): 323–324 15313987 10.1503/cmaj.1031298 PMC509040

[bib27] Mor G, Visintin I, Lai Y, Zhao H, Schwartz P, Rutherford T, Yue L, Bray-Ward P, Ward DC (2005) Serum protein markers for early detection of ovarian cancer. Proc Natl Acad Sci USA 102(21): 7677–7682 15890779 10.1073/pnas.0502178102 PMC1140439

[bib28] Oei AL, Massuger LF, Bulten J, Ligtenberg M, Hoogerbrugge N, de Hullu JA (2006) Surveillance of women at high risk for hereditary ovarian cancer is inefficient. Br J Cancer 94: 814–819 16495917 10.1038/sj.bjc.6603015 PMC2361371

[bib29] Olivier RI, Lubsen-Brandsma MA, Verhoef S, van Beurden M (2006) CA125 and transvaginal ultrasound monitoring in high-risk women cannot prevent the diagnosis of advanced ovarian cancer. Gynecol Oncol 100(1): 20–26 16188302 10.1016/j.ygyno.2005.08.038

[bib30] Peto J, Collins N, Barfoot R, Seal S, Warren W, Rahman N, Easton DF, Evans C, Deacon J, Stratton MR (1999) Prevalence of BRCA1 and BRCA2 gene mutations in patients with early-onset breast cancer. J Natl Cancer Inst 91(11): 943–949 10359546 10.1093/jnci/91.11.943

[bib31] Rebbeck TR, Friebel T, Wagner T, Lynch HT, Garber JE, Daly MB, Isaacs C, Olopade OI, Neuhausen SL, van ’t Veer L, Eeles R, Evans DG, Tomlinson G, Matloff E, Narod SA, Eisen A, Domchek S, Armstrong K, Weber BL (2005) Effect of short-term hormone replacement therapy on breast cancer risk reduction after bilateral prophylactic oophorectomy in BRCA1 and BRCA2 mutation carriers: the PROSE Study Group. J Clin Oncol 23(31): 7804–7810 16219936 10.1200/JCO.2004.00.8151

[bib32] Rebbeck TR, Lynch HT, Neuhausen SL, Narod SA, Van’t Veer L, Garber JE, Evans G, Isaacs C, Daly MB, Matloff E, Olopade OI, Weber BL (2002) Prophylactic oophorectomy in carriers of BRCA1 or BRCA2 mutations. N Engl J Med 346(21): 1616–1622 12023993 10.1056/NEJMoa012158

[bib33] Scheuer L, Kauff N, Robson M, Kelly B, Barakat R, Satagopan J, Ellis N, Hensley M, Boyd J, Borgen P, Norton L, Offit K (2002) Outcome of preventive surgery and screening for breast and ovarian cancer in BRCA mutation carriers. J Clin Oncol 20(5): 1260–1268 11870168 10.1200/JCO.2002.20.5.1260

[bib34] Stirling D, Evans DG, Pichert G, Shenton A, Kirk EN, Rimmer S, Steel CM, Lawson S, Busby-Earle RMC, Walker J, Lalloo FI, Eccles DM, Lucassen AM, Porteous ME (2005) Screening for familial ovarian cancer: failure of current protocols to detect ovarian cancer at an early stage according to the international Federation of gynaecology and obstetrics system. J Clin Oncol 23(24): 5588–5596 16110018 10.1200/JCO.2005.05.097

[bib35] Tailor A, Bourne TH, Campbell S, Okokon E, Dew T, Collins WP (2003) Results from an ultrasound-based familial ovarian cancer screening clinic: a 10-year observational study. Ultrasound Obstet Gynecol 21(4): 378–385 12704748 10.1002/uog.65

[bib36] Tinley ST, Houfek J, Watson P, Wenzel L, Clark MB, Coughlin S, Lynch HT (2004) Screening adherence in BRCA1/2 families is associated with primary physicians’ behavior. Am J Med Genet 125(1): 5–11 10.1002/ajmg.a.20431 14755459

[bib37] Vasen HF, Tesfay E, Boonstra H, Mourits MJ, Rutgers E, Verheijen R, Oosterwijk J, Beex L (2005) Early detection of breast and ovarian cancer in families with BRCA mutations. Eur J Cancer 41(4): 549–554 15737559 10.1016/j.ejca.2004.10.029

[bib38] Wainburg S, Husted J (2004) Utilization of screening and preventive surgery among unaffected carriers of a BRCA1 or BRCA2 gene mutation. Cancer Epidemiol Biomarkers Prev 13(12): 1989–1995 15598752

[bib39] Whittemore AS, Balise RR, Pharoah PD, DiCioccio RA, Oakley-Girvan I, Ramus SJ, Daly M, Usinowicz MB, Garlinghouse-Jones K, Ponder BAJ, Buys S, Senie R, Andrulis I, John E, Hopper JL, Piver MS (2004) Oral contraceptive use and ovarian cancer risk among carriers of BRCA1 or BRCA2 mutations. Br J Cancer 91(11): 1911–1915; www.nvog.nl. 2006 15545966 10.1038/sj.bjc.6602239 PMC2410144

[bib40] Zhang Z, Bast RC, Yu Y, Li J, Sokoll LJ, Rai AJ, Rosenzweig JM, Cameron B, Wang YY, Meng X, Berchuck A, Haaften-Day van C, Hacker NF, Bruijn de HW, Zee van der AG, Jacobs IJ, Fung ET, Chan DW (2004) Three biomarkers identified from serum proteomic analysis for the detection of early stage ovarian cancer. Cancer Res 64(16): 5882–5890 15313933 10.1158/0008-5472.CAN-04-0746

